# Exploring the care experiences of hemodialysis nurses: from the cultural sensitivity approach

**DOI:** 10.1186/s12912-023-01678-y

**Published:** 2024-01-02

**Authors:** Hsu Jui-Chin, Chung Fen-Fang, Lee Tso-Ying, Wang Pao-Yu, Lin Mei-Hsiang

**Affiliations:** 1 Hemodialysis Room, Cheng Hsin General Hospital, Taipei, Taiwan, R.O.C.; 2 Department of Nursing, Chang Gung University of Science and Technology, Tao-Yuan, Taiwan, R.O.C.; 3 Nursing research center of Taipei Medical University Hospital, Taipei, Taiwan, R.O.C.; 4 Department of Nursing, MacKay Junior College of Medicine, Nursing and Management, Taipei, Taiwan, R.O.C.; 5 EdD School of Nursing, National Taipei University of Nursing and Health Sciences, No. 365, Mingde 1st Rd. Beitou Dist, Taipei, Taiwan, R.O.C.

## Abstract

**Background:**

Culturally sensitive care has been identified as a best-practice approach for improving health outcomes. Hemodialysis patients require culturally sensitive care because it involves totally changing their previous life. The purpose of this study was to explore the subjective experiences of hemodialysis nurses in providing culturally sensitive care to hemodialysis patients.

**Methods:**

A qualitative study was carried out in the hemodialysis center of a teaching hospital in northern Taiwan. Purposive sampling and semi-structured interview guidelines were employed to interview 23 hemodialysis nurses. The interviews were recorded and transcribed verbatim, and the resulting data were analyzed and summarized using content analysis by constant comparative methods.

**Results:**

Hemodialysis nurses exhibited the characteristics for delivering culturally sensitive care, which comprised five aspects: finding the true meaning of the behavior of the participants, recognizing and honoring individual psychological states, culturally sensitive communication in line with patients’ values, customizing care content through cultural transformation strategies, and empowerment rather than prohibition.

**Conclusions:**

The findings of this study on the culturally sensitive care provided by hemodialysis nurses can be utilized by nursing educators and administrators as a reference to develop and enhance the nursing education related to culturally sensitive care for hemodialysis professionals.

## Background

Cultural sensitivity is broadly recognized as the knowledge, skills, attitudes and beliefs that enable people to work well with, respond effectively to, and be supportive of people in cross-cultural settings [1]. Being culturally sensitive means to have cultural knowledge, and to meet patients with an open, and person-oriented attitude [2]. Also, patient-centred, culturally sensitive care has been identified as a best-practice approach for improving health outcomes and reducing health disparities [3]. Studies have revealed that the cultural background of HD patients can affect their compliance with dialysis treatment [4, 5]. Building cultural sensitivity requires self-awareness, along with the ability and willingness to examine one’s ethnocultural, attitudes, beliefs, and behavior [6]. A patient’s cultural values, beliefs, and practices are an important part of holistic nursing care [7]. Culturally adequate nursing care enables individuals to benefit from health services, and reduces inequalities in access to health services through the integration of the patient’s cultural beliefs into patient care [8].

Also, according to Claramita et al. [9] mentioned that improving nurses’ cultural sensitivity could improve quality of care and increase patient satisfaction. Foronda [10] conducted a concept analysis of cultural sensitivity which findings included the attributes of knowledge, consideration, understanding, respect. The consequences were effective communication, effective intervention, and satisfaction. Majumdar et al. [11] postulated that culturally sensitive care has also been described as care in which health care providers offer services in a manner that is relevant to patients’ needs and expectations. According to Bennett [6], intercultural sensitivity is not “natural” to any single culture, the development of this ability demands new awareness and attitudes. Therefore, building cultural sensitivity requires self-awareness and a willingness to adapt to people’s cultures, attitudes, beliefs and behaviors that differ from one’s own [6, 12].

HD patients require culturally sensitive care because it involves totally changing their previous life. End Stage Renal Disease (ESRD) represents the end stage of chronic kidney disease, and hemodialysis (HD) is presently the most common treatment for ESRD [13]. Chronic kidney disease has become a prevalent public health issue worldwide, with increasing incidence rates and significant mortality rates in many regions [14]. Overall, patients with ESRD generally have a stable disease course and are not immediately in life-threatening danger as long as they adhere to medical protocols [15]. However, prolonged HD treatment can impose significant physiological, psychological, social, and other types of discomfort and stress on patients, in addition to the restrictions on their daily life. Patients facing uncertain futures may feel that they are a burden to others or their families [16]. In addition to having to visit the dialysis center three times a week, HD patients are faced with restrictions on their daily routines, work schedules, and leisure activities. These limitations prevent them from maintaining their pre-dialysis lifestyle, and they may feel hopeless about their future. Under these circumstances, patients may contemplate ending their lives to avoid being a burden on their families, leading to a rise in the proportion of those with depression [17, 18]. However, there were few studies conducted with cultural sensitivity care for HD patients.

Chang et al. [19] studied factors affecting diet-related quality of life among hemodialysis patients. They found that diet in patients undergoing hemodialysis was still strongly influenced by culture. Just as Chinese people have always held a belief that animal organs correspond to human organs. For example, some HD patients think that their kidneys are damaged, so they can eat pig’s kidney to enhance their renal function [20]. Hence, nurses must be willing, aware and knowledgeable and become experienced in caring for individuals of various cultural backgrounds so they can provide culturally sensitive [21]. Improving the multicultural competence of nephrology professional nurses and establishing culturally sensitive care can help eliminate racial and ethnic disparities in patients with chronic kidney disease, thereby reducing health inequalities [22].

Taiwan has the highest incidence and prevalence of ESRD worldwide [13]. Although the health insurance system provides good dialysis treatment for patients with chronic kidney disease, offering them a chance of survival, their mortality rate is higher than that of the general population due to the negative impact of chronic kidney disease, comorbidities, and dialysis treatment [13]. In clinical nursing practice, it is essential to emphasize the effectiveness of the patient’s dialysis treatment and the interpretation of test results while also providing health education on diet and disease management based on the test data. Furthermore, cultural sensitivity is an ongoing awareness of cultural differences and similarities among populations [11]. Drawing from the literature above, it can be inferred that providing culturally sensitive and patient-centered nursing care, in addition to advanced professional training in HD, can enhance the quality of dialysis care in clinical settings. Although Taiwan is an island, it is important to acknowledge the presence of cultural differences among various ethnic groups and generations. Therefore, this study primarily focused on the clinical HD personnel trained in Taiwan to address the following questions: What efforts have been made in terms of cultural care, and how can further progress be achieved? By using the qualitative research results, information about the cultural care experiences of HD nurses can be obtained, enabling the provision of appropriate healthcare for future patients and improving the quality of clinical care.

## Methods

### Design and participants

This study adopted the qualitative research method, and face-to-face semi-structured interviews were conducted from April to August 2022. This study followed the consolidated criteria for reporting qualitative research (COREQ) checklist to ensure the quality of research [23].

### Participants

This study adopted purposive sampling to enroll participants at a medical center in northern Taiwan. The inclusion criteria were nurses who had worked at the hospital for more than one year, had been working in HD clinical practice for at least three months, and were willing to take part in this research. The exclusion criteria were nurses who were diagnosed with cancer or depression. Data collection stopped when data saturation was achieved, as reflected in the data analysis. Data saturation was used to determine the final number of participants [24]. We stopped conducting interviews when no new themes were emerging, after theoretical saturation had been achieved [25]. After the 23rd interview, there were no new themes generated from the interviews. Therefore, it was deemed that the data collection had reached a saturation point.

### Data collection

In this study, interviews were arranged after obtaining the consent of the participants who met the inclusion criteria. The interview location was selected based on the principle that the interviewees could freely describe their experiences. In-depth interviews were conducted to collect data for this study using interview guidelines that included the following questions: “What are the main issues that you encountered in your experience of caring for hemodialysis patients?” “What do you think of the changes in the lives of these patients after they started hemodialysis?” “How do you think the hemodialysis patients’ different cultural backgrounds?” and “How do you inform patients about the positive aspects of undergoing hemodialysis?” Audio-recorded interviews ranged from 60 to 90 min and were immediately transcribed upon completion by a research assistant, who had no prior contact with respondents.

### Data analysis

This study used content analysis by constant comparative [26]. The analysis involved the following steps: (1) within 72 h of the interview, the interview recording was transcribed verbatim into a narrative record with the consideration of the observed non-verbal behavior in the summary notes; (2) the transcript was carefully reviewed several times to obtain an overall understanding of the interview; (3) the verbal statements of the participants were systematically analyzed and categorized. The researchers listened to the interview content several times and then created a summary of the interview with key ideas or concepts; (4) after reading and conceptualizing the text over and over again, we discussed the themes and ensured that they were inherent in the texts; (5) we identified the essential themes, classified them, and determined the terms that were most strongly and consistently related to the phenomenon. This process included both inductive and deductive reasoning activities [27]. Finally, all of the presented subthemes were integrated to establish a complete context of the culturally sensitive care experiences of HD nurses. The data analysis of this study was coded by the first author and the corresponding author. The key lines and condensed meaning units in the text were marked, followed by coding to generate the initial code. Similar codes were grouped together to create subcategories and categories, which in turn formed topics. Data analysis was conducted throughout the entire data collection. Other authors read samples of the coding interviews to examine the coding. All authors discussed the assigned code multiple times until a consensus was reached.

### Rigor

In terms of rigor, four indicators developed by Lincoln and Guba [28] suitable for qualitative research models were adopted. (1) Credibility: in addition to the qualitative research training and experience that the project team had, to ensure credibility, during the data analysis period, the interviewers spent an extended period of time at the study site to gain a deeper understanding of the cultural context of the participants and build trusting interpersonal relationships with them. Peer debriefing was used to discuss and adjust the results several times, and an expert with qualitative research experience checked the accuracy of the analysis. Additionally, member checks were conducted by inviting three participants to review the results to test whether the results truly reflected their experiences. (2) Transferability: the investigators faithfully transferred the rich descriptions of the subjective experiences from the participants into written data and avoided the addition of personal opinions, thus enabling the findings to be applicable to other clinical settings. (3) Dependability: the entire process of the interview was recorded, and the interviewers personally transcribed the recordings into transcripts for further analysis. Finally, two investigators re-examined the degree of consistency between the recordings and the transcripts to enhance the dependability. (4) Conformability: once the credibility, transferability, and dependability of the research were achieved, the conformability of the study was naturally achieved.

### Ethical considerations

In this study, data collection started after the research protocol was approved by the Institutional Review Board (IRB) of the teaching hospital in northern Taiwan (CHGH-IRB: (887)110 A-33). Before data collection, the investigator first explained the study objective and the procedures to the potential participants and then took the initiative to inform them that they had the right to withdraw from the study at any time. Interviews were conducted only after the participants provided their informed consent and signed the consent form.

## Results

A total of 23 HD nurses were interviewed for this study. The detailed characteristics of the participants are presented in Table 1. Their mean HD nursing experience was 14.11 years, with a range of 2.25 to 25.83 years. 4.34% had the experience of attending cultural courses (*n* = 1), and 43.47% had the experience of caring for foreigners (*n* = 10). Nearly half (*n* = 11, 47.83%) of the participants had a professional nursing competence level of N2. In Taiwan, the nursing competence level is based on the period of clinical experience and competence, with less than 12 months nurses as level N (Ns), greater than 12 months as level 1 nurses (N1s, responsible for basic nursing), 2 years or more as level 2 nurses (N2s, critical care nursing), 3 years or more as level 3 nurses (N3s, in charge of education and holistic nursing), 4 years or more as level 4 nurses (N4s, responsible for research and specialized nursing). This system has been widely adopted by medical centers for many years in Taiwan [29]. The levels are differentiated according to qualitative and quantitative indicators, including clinical competence, professional excellence, and educational attainment [30]. Nursing ladder management has profound influences on improving nurses’ qualifications, promoting their career advancement, augmenting care outcomes, and enhancing the rate of patient satisfaction [31].

**Table 1:** Characteristics of the participants

participants	Age (years)	Marital status	Educational level	Years of nursing experience	Nursing competency level	Experience in caring for foreigners	Experience of attending cultural courses
A	39	Single	College	18	N3	yes	no
B	47	Single	University	28	N2	no	no
C	40	Married	University	15	N3	no	no
D	39	Married	College	20	N1	yes	no
E	43	Married	University	23	N2	no	no
F	51	Married	University	29	N3	no	no
G	46	Married	University	23	N2	no	no
H	48	Married	College	25	N2	yes	no
I	43	Single	Master	15	N1	yes	yes
J	43	Married	University	25	N3	yes	no
K	54	Married	University	35	N2	no	no
L	46	Married	College	25	N2	no	no
M	35	Single	University	12	N3	no	no
N	53	Single	College	28	N2	no	no
O	50	Married	University	30	N1	yes	no
P	45	Single	University	23	N2	yes	no
Q	47	Single	College	26	N	yes	no
R	44	Married	College	15	N	no	no
S	46	Married	College	26	N2	no	no
T	32	Single	College	12	N1	yes	no
U	37	Single	University	8	N1	yes	no
V	32	Married	University	11	N2	no	no
W	41	Single	University	17	N2	no	no

Through in-depth interviews and data analysis, the five key qualities that HD nurses should have for providing culturally sensitive care emerged, including finding the true meaning of the behavior of the participants, recognizing and honoring individual psychological states, culturally sensitive communication in line with patients’ values, customizing care content through cultural transformation strategies, and empowerment rather than prohibition. Overall, “respect” was the essence of the care provided by the HD nurses, with “empowerment rather than prohibition” serving as the core of their approach in the process of providing culturally sensitive care. Overall, the culturally sensitive experiences of HD nurses caring for HD patients were divided into five main themes and 13 sub-themes as shown in Table 2.

**Table 2:** Main themes and sub-themes of the study

Main themes	sub-themes
Finding the true meaning of the behavior of the participants	• Understanding that refusal behavior reflects unacceptance
	• Understanding that tantrums are a manifestation of wanting to be cared for
Recognizing and honoring individual psychological states	• Respecting and allowing the state of readiness to emerge
	• Respecting the patient’s “no change” phase prior to transitioning to “acceptance”
Culturally sensitive communication in line with patients’ values	• Guiding patient to accept treatment using their beliefs in fate
	• Fostering awareness of the effectiveness of dialysis
	• Promoting a shift in perspective towards the positive meaning of dialysis
	• Leveraging the influence of “relatives”
Customizing care content through cultural transformation strategies	• Explaining information patiently and repeatedly in a more acceptable way
	• Reminding patients of information with a relaxed manner and non-blaming words
	• Borrowing physician’s authority
Empowerment rather than prohibition	• Not interfering with patients’ coping behavior
	• Giving specific responses to concerns

### Finding the true meaning of the behavior of the participants

For Taiwanese patients, the term “dialysis” carries the connotation of “kidney function deterioration” and is perceived as a distressing event synonymous with “life being completely ruined.” Consequently, when a patient is informed about the necessity of undergoing HD, they initially struggle to accept the reality of lifelong dialysis. This often leads to such behaviors as rejection and anger.

#### Understanding that refusal behavior reflects unacceptance

HD nurses could detect through observation and further clarification that refusal behavior in individual patients may be a manifestation of their unacceptance of the disease and treatment.

Participant A mentioned that during the initial health education of patients, young patients may directly express their reluctance by stating, “*I don’t want to discuss this issue.*” It is possible that they are initially unable to accept the reality of their situation. The participant shared an encounter with an older patient who, although capable of hearing, would say, “*I don’t understand Taiwanese language, and I can’t hear you*,” when faced with the issue of HD. It was evident that he simply did not want to confront the matter under discussion. In response, the participant decided to refrain from discussing the topic at that moment and planned to revisit it later to gauge if the patient would be receptive. It is important to approach this slowly, allowing the patient to progress at their own pace. The participant stated that when initially informing the patient, “*You need dialysis*,” the patient cannot accept it whatsoever.

#### Understanding that tantrums are a manifestation of wanting to be cared for

HD nurses also understood that “tantrums” may be a patient’s way of expressing a need for care, so they empathized with the patient and accepted the behavior.

Participant W shared that elderly patients sometimes display reluctance in accepting dialysis, even expressing emotions through tears and by saying, “*I don’t want dialysis! Don’t torture me like this!*” Participant W stated, “*patient communication should be approached gradually, recognizing that the patient’s initial resistance stems from their difficulty with accepting the situation and is not an intentional tantrum. Older patients, much like children, often seek additional care from family members. They simply want compassionate attention.*”

### Recognizing and honoring individual psychological states

In Taiwan, many older adults hold the belief that “dialysis treatment is necessary until death” and perceive “dialysis” as a “terminal disease.” Consequently, when patients are informed about the deterioration of their kidney function and the necessity of lifelong HD, it is equivalent to being “sentenced to death,” which requires time to come to terms with the situation.

#### Respecting and allowing the state of readiness to emerge

HD nurses recognized that each individual requires varying amounts of time to process their emotions. Therefore, it is crucial to show respect and exercise patience, allowing the patient’s mood to reach a state of readiness in their own time and circumstances.

For example, Participant B stated, *“…After all, dialysis is a reality that must be faced. Patients should not be forced to say yes immediately or accept dialysis happily…”*

Similarly, participant H stated, *“…if the patient is depressed, wait until he is more emotionally stable, or when he is able to respond, and then… continue to communicate. You can’t rush into a conversation about dialysis.”*

#### Respecting the patient’s “no change” phase prior to transitioning to “acceptance”

Most Taiwanese people have a misconception that certain foods should be avoided during the “dialysis” period. Taiwanese culture traditionally emphasizes the concept that “food is of paramount importance to people” and holds a belief in the practice of “food therapy.” Consequently, the notion of “nothing to eat” as a result of word-of-mouth is often believed and circulated by patients regarding specific dietary restrictions during dialysis. This misconception leads to the idea that “if you don’t eat and lack energy, you can only await death.” As a result, medical staff in the dialysis unit face the challenge of effectively communicating with patients and providing accurate information. Prior to communication, HD nurses prioritized respecting the patient’s attitude for “no change” until their acceptance of the necessary adjustments.

As Participant G stated, *“It’s not that patients don’t understand; it’s that they don’t want to listen. They don’t even want to cooperate… It’s understandable. They come for dialysis three times a week, and I’ve been telling them the same thing every time; perhaps what they want to hear is that I care about them.” Patients have even said, “I am so old. If I can’t drink coffee or eat anything I like, what’s the point of living?”*

### Culturally sensitive communication in line with patients’ values

In addition to respecting and allowing individual patients to come to terms with the reality of HD, HD nurses employed various strategies to communicate effectively using language that aligns with the patient’s cultural customs and values. They assisted patients in adjusting and accepting the necessity of HD treatment.

#### Guiding patient to accept treatment using their beliefs in fate

Certain individuals in Taiwan hold a belief in “fate,” much like the Buddhist perspective asserting that “everything has a destiny and cannot be coerced.” Consequently, some nurses employ the “fate theory” to assist patients in acknowledging and accepting the reality of undergoing dialysis.

As Participant A explained, “…*Sometimes I tell the patient, ‘You are just unlucky that your condition deteriorated, and you need dialysis…’ as a way to help patients accept their conditions.”*

#### Fostering awareness of the effectiveness of dialysis

HD nurses assisted patients in accepting reality by guiding them to recognize that dialysis can lead to an improvement in physical discomfort symptoms, thereby reinforcing the notion of the “effectiveness of dialysis.”

Participant A shared, *“…The patient was very uncomfortable when he first came for HD… After receiving HD a few times… I asked him: ‘How are your discomforts after having HD?’ The patients would tell me, ‘They have really improved.’ He might also say, ‘my breathlessness is improved,’ ‘My blood pressure is not as high, and my appetite is better,’ or ‘Nausea has improved…’”*

#### Promoting a shift in perspective towards the positive meaning of dialysis

Patients who undergo regular dialysis in hospitals, typically lasting 3–4 h each time, often experience a sense of confinement and hopelessness as they perceive dialysis as the sole remaining aspect of their lives. HD nurses play a crucial role in encouraging a shift in perspective to recognize the positive significance of dialysis.

For instance, Participant E motivated patients by highlighting that “dialysis” carries a positive meaning of enhancing the quality of life: *“Some patients who have recently started HD are resistant because they have to visit the hospital every week for a 3- to 4-hour dialysis session. I help them to see that this weekly visit is similar to rehabilitation therapy, which can prolong and improve the quality of their life, unlike other diseases that cannot be treated.“.* Some nurses encouraged patients to view the repeated dialysis process as “going to work.”

Participant Q shared, *“I told the patient… you can imagine that you are working in the dialysis room. You come here for four hours, enjoy the air conditioning for free, and the doctors and nurses take care of you wholeheartedly.”*

Moreover, some HD nurses guided patients to find meaning in their HD treatment.

For example, participant T shared the experience of caring for elderly patients: *“I would ask senior dialysis patients, ‘Do you have any grandchildren? Are they cute?’ They would say ‘yes’ most of the time, and then I would ask, ‘Would you like to see them go to elementary school?’ They would definitely say ‘yes,’ and I would follow up by saying that if they adhere to dialysis treatment, they will have that opportunity.”*

Whether through the theory of fate or by attributing positive meaning, the aim was to assist patients in accepting the realities of HD to the fullest extent possible and to foster their cooperation.

#### Leveraging the influence of “relatives”

Taiwanese culture values “family relationships” greatly. In addition to the support from patient support groups, HD nurses skillfully utilized the influence of “relatives.”

Participant N explained that communication about accepting HD with patients could be challenging: *“I would communicate with the family members first… because they seem to be more receptive. I then ask the family members to communicate with the patient gradually. Some patients would say, ‘Don’t tell me, just talk to my children…’ because they can’t accept the reality…”* Based on the interview data, it is evident that the support of family members plays a crucial role as a driving force behind the active cooperation of HD patients in Taiwan.

### Customizing care content through cultural transformation strategies

The cultural transformation strategy entails nurses delivering care tailored to patients’ cultural background and individual circumstances in a manner that patients can accept. This approach aims to facilitate behavior change and promote the acceptance of health education content. Patients undergoing long-term HD often experience resistance towards dietary restrictions, limitations on water intake, and various daily life activities, compounded by frequent reminders from their surroundings. Culturally sensitive HD nurses modified the health education content to align with the patient’s cultural background and personal situation. This approach helped alleviate patient resistance and enhanced the effectiveness of health education.

#### Explaining information patiently and repeatedly in a more acceptable way

Professional nurses demonstrated patience and repeatedly provided explanations to patients, presenting information in a manner that is different yet acceptable. For instance, certain Taiwanese individuals consider duck eggs and carambola to be “toxic” foods, leading to a reluctance to consume carambola.

Thus, participant A stated, *“When teaching first-time dialysis patients, I tell them that there is only one food they must not eat, and that is star fruit. They can eat any other foods they want but in moderation. If I say they cannot eat oranges, bananas, etc., the patient may feel that they are already confined to the hospital because of HD, and these additional dietary restrictions make life meaningless. Some patients may even say, ‘It’s better to just die…’”*

#### Reminding patients of information with a relaxed manner and non-blaming words

Patients may occasionally deviate from their dietary restrictions due to “cravings” or a “lack of interest,” leading to poor blood test results or discomfort during dialysis. HD nurses used relaxed and non-accusatory language to remind them of certain information.

Participant W explained, *“Senior HD patients may feel that they have been receiving HD treatments for a long time and have more experience than the nurses… Therefore, I would conduct health education in a humorous manner because if I point out their errors directly, they may resist even more.”*

#### Borrowing physician’s authority

Taiwanese individuals, especially older adults, highly value their reputation and hold a deep respect for doctors. They often view the words of doctors as “sacred decrees.” In cases where patients’ self-management was severely lacking and had a significant impact on their health, HD nurses would involve doctors in a timely manner to encourage compliance with medication and diet.

Participant J described this approach, saying, *“When necessary, we ask the doctor to explain to the patient. Patients tend to be more compliant with the authority of a doctor…”*

### Empowerment rather than prohibition

HD is a long journey. Culturally sensitive HD nurses provided personalized care, presented options rather than imposed restrictions, addressed concerns directly, respected patients’ behavioral reactions to the treatment, and empowered patients to make decisions.

#### Not interfering with patients’ coping behavior

Establishing a therapeutic relationship with the individual is an increasingly common concept in clinical care. In our study, respecting patients’ behavioral responses to treatment without blocking them was an important aspect of “empowerment” in providing care. Taiwanese patients believe that a clean environment can promote well-being and aid in the restoration of health, attributing their need for dialysis to the presence of “toxins in urine.”

As participant P highlighted, *“Some patients may feel that dialysis patients are dirty, so they disinfect the hospital bed before lying on it to feel more secure. HD nurses cannot block the patient, nor should they try to stop patients from doing so.”*

#### Giving specific responses to concerns

Nurses working in dialysis units face complex and highly challenging clinical situations. Further, responding specifically to patients’ concerns is a form of empowerment care. Taiwanese people hold the belief that blood may encompasses the vital energy of the human body and is a possible fundamental part of “qi.” (pronounced as “chee”).

Participant C shared that patients often express their worries to her, such as about the preciousness of blood, as the Hb level of dialysis patients may drop easily. To address this concern, Participant C shared that she would ensure to the patient that all of the blood in the dialysis machine would be returned to the patient’s body before ending the session.

## Discussion

This study aimed to explore the care process and cultural experiences of HD nurses. Five main themes emerged: identifying genuine behaviors, demonstrating patience as patients adjust to dialysis, facilitating patient acceptance of dialysis, providing care from a diverse perspective, and empowering patients rather than imposing restrictions. The findings indicated that some of the interpretations of cultural care by HD nurses were consistent with those of previous studies. The results also revealed unique experiences specific to the local socio-cultural context.

As chronic kidney disease progresses, patients often need to make lifestyle changes, which can result in considerable psychological stress [32]. In the process of caring for HD patients, the participants of this study observed and clarified that the “refusal” of dialysis behavior was a manifestation of unacceptance exhibited by the patients. As indicated by Ratti et al. [33], new HD patients often experience negative emotions, such as anger, frustration, and pessimism, due to the impact of the disease and the discomfort associated with the treatment process. However, during the process of patient care, HD nurses observed that the “refusal” behavior displayed by HD patients was an expression of their difficulty with accepting their situation. According to Campinha-Bacote [34], cultural sensitivity is the respect and appreciation of clients’ health beliefs and practices. Hence, participants recognized that such “tantrum” behavior was a way for patients to seek care and attention which is seems to be an overinterpretation. Culture emerges from the interplay of an individual’s social background and their engagement in role-playing, encompassing unique characteristics [35]. As chronic kidney disease progresses, patients need to make lifelong changes in their eating habits [36]. The participants of this study recognized that each patient required a different amount of time to cope with the emotional impact of needing lifelong HD. Therefore, they were willing to accompany the patients and wait for them. Most patients with chronic kidney disease often experience daily stress due to treatment regimens, medications, dietary and fluid restrictions, and lifestyle changes [37]. In this study, the participants continued to communicate with and accompany patients while waiting for their willingness to undergo dialysis, respecting patients’ “resistance to change” behavior. These results are consistent with those of Garvey et al. [15], which emphasized that culturally sensitive HD nurses can foster an environment that respects cultural health disparities and provides effective healthcare services.

HD is the most common treatment in renal replacement therapy (RRT) [16]. Patients who undergo HD typically have complex medical histories, are required to take multiple medications, and must adhere to dietary restrictions. Therefore, patients require comprehensive education about their RRT options. Adequate communication between healthcare professionals and patients is crucial when assisting patients in accepting dialysis [32]. In this study, the participants recognized that effective communication, employing language aligned with the patient’s cultural conventions and values, played a pivotal role in helping patients accept the realities of HD. In this study, the participants guided patients to find meaning in their life with dialysis, which aligns with previous research on the resilience of HD patients. Patients were willing to find a sense of purpose and overcome the challenges of HD and were inclined to seek out supportive relationships and networks [37]. Furthermore, drawing upon the Chinese emphasis on “family relationships,” the participants of this study effectively leveraged the influence of “relatives” to encourage active patient cooperation with HD treatment. These results are consistent with those of multiple studies indicating that nurses have the closest relationship with patients and families, serving as evaluators, information providers, supporters, and educators. They respect and support all stakeholders after understanding their preferences and how they affect one another [32, 38]. Moreover, through culturally sensitive communication, the participants of this study were able to encourage patients to “change their perspective” on receiving dialysis and utilized the support of kidney disease support groups and family members to provide patient support. These findings are consistent with the results of a study by Claramita et al. [9], which highlighted the importance of culturally sensitive communication in demonstrating understanding and respect for individuals, leading to increased satisfaction in patients and their families.

Factors that impact patients’ acceptance of HD include necessary lifestyle changes to their diet, the time commitment for HD therapy, and strict medication and dietary control [39]. The participants of this study delivered care in a manner that was accepted by patients, taking into account the patients’ cultural background and personal circumstances. They offered reminders using casual and non-blaming language. These results support the findings of Hayes et al. [40], showing that HD nurses develop long-term relationships with patients to provide individualized care in a technically complex work environment. Furthermore, within Taiwan’s medical-seeking culture, patients regard doctors’ words as akin to “sacred decrees.” Recognizing this belief, the participants of this study effectively utilized the authority of doctors to encourage HD patients’ adherence to dietary and medication requirements. This phenomenon has also been noted in previous studies. Nephrology nurses should establish culturally sensitive nursing practices and provide individualized patient education to promote quality of care and continuity of care, in addition to maintaining communication with interdisciplinary teams and relevant personnel [22, 41].

HD is a long journey. In this study, participants provided care that was acceptable to patients, empowered patients by giving them the right to choose, replaced prohibition with options, and accepted the patients’ behavioral reactions to the treatment. These findings align with the World Health Organization ‘s advocacy for patients to have the right to make decisions about their own health. Patient empowerment is a concept that was introduced to allow patients to shed their passive role and play an active part in the decision-making process about their health and quality of life [42]. The participants of this study indicated that within Taiwanese culture, some patients undergoing HD for the first time perceived dialysis patients as having the “uremia” disease. They considered that their urine was toxic, leading to a sense of being unclean. Additionally, certain older adults had the misconception that carambola was poisonous. The star fruit (Averrhoa carambola) is widely prevalent in tropical regions across the globe, with significant consumption levels in Asia, Central America, and South America. [43, 44]. As per Yasawardene et al. [45], the star fruit (Averrhoa carambola) contains oxalic acid and caramboxin. The consequences of star fruit ingestion are primarily associated with nephrotoxicity and neurotoxicity. Several studies have extensively documented star fruit-induced nephrotoxicity, specifically presenting as acute kidney injury and neurological impairment, particularly in individuals with pre-existing renal conditions [45–48]. Nevertheless, several studies have suggested that neurotoxicity is not commonly reported among individuals with previously normal renal function [43, 44]. Therefore, healthcare professionals should counsel patients with chronic kidney disease or those undergoing dialysis to refrain from consuming star fruit [49].

Simultaneously, certain HD nurses in this study highlighted that patients undergoing dialysis frequently discuss the significance of blood and express apprehensions about diminishing hemoglobin levels. The physiological changes observed in patients due to dialysis in this study may align with the perspective of Cozzolino et al. [50], who postulated that dialysis patients often experience complications such as anemia, cardiovascular problems, and bone lesions. Moreover, the extraction of blood during dialysis has the potential to cause deficiencies, resulting in sensations of fatigue and weakness [51]. In traditional Chinese medicine (TCM), blood plays various crucial roles beyond its recognized physiological functions in Western medicine [52]. Furthermore, in TCM, any symptom of physical, emotional, or psychological illness is believed to stem from an imbalance in what is referred to as the internal energies or Qi in Chinese [53]. Qi is intricately woven into Chinese culture, distilled to its essence as “vital energy” that animates all things-without Qi, there is no life [54]. A previous study has affirmed the connection between Qi and blood, offering scientific support for the principles of Qi in TCM [55]. In the amalgamation of blood and Qi, forming blood-Qi, Qi adopts the life-force vitality of blood as a carrier of oxygen and nutrients, which are also perceived as manifestations of Qi [54]. Additionally, as highlighted by Ong [54], blood-Qi, acting as a surrogate for Qi, can be accessed through blood perfusion for valuable medical diagnostics. This is particularly evident in impaired tissues, and blood perfusion measurements are commonly utilized in the diagnosis and management of various medical conditions. As highlighted by Levy et al. [56], inadequate perfusion may underlie a substantial portion of the tissue and organ dysfunction associated with chronic conditions such as hypertension and diabetes mellitus. Therefore, based on the literature mentioned above, for dialysis patients, blood may encompass the vital energy of the human body and is a possible fundamental part of “qi”. Considering these clinical HD care issues, the participants in this study provided health education to patients in an acceptable manner by responding specifically to their concerns and gaining insight into their ability to manage their own lives. The findings of this study align with a study by Shih et al. [57], which suggested that by considering patients’ sociocultural context and relationships and respecting their individual preferences, needs, and values in a relationship with trust, care, and sincerity, healthcare professionals can ensure that clinical decisions are made in accordance with patients’ own values.

Among the participants of this study, A small number of the participants had experience in attending cultural courses, while a little less than half had experience in caring for foreigners. Previous research has shown that the experience of nursing staff in caring for foreigners has an impact on their cultural sensitivity [21]. In addition, Chang et al. [58] reported that “conducting or attending cross-cultural activities” and “having friends from different cultural backgrounds” positively affect cultural sensitivity and improve an individual’s cultural competence. Therefore, future studies should further investigate the effect of caring for foreigners and receiving relevant training on the provision of culturally sensitive care to HD patients. While the focus of this study was to explore the culturally sensitive care process and experiences of HD nurses, the data collected in this study reflected the views of HD nurses, which may contain subjective biases. Therefore, caution should be exercised when interpreting the results, particularly for individuals with different life experiences and backgrounds.

### Limitations

The study was conducted at one teaching hospital in Taiwan. This might have limited the potential of generalizing our findings to other contexts, and interpretation of the findings should be done with caution. However, this study explored the cultural sensitivity of HD nurses, giving us a unique understanding of and insight into the role of HD nurses in caring for HD patients. This study focused on “culturally sensitive” care. Participants’ cultural and religious beliefs were not captured in this study. Future research should take this information into consideration. Finally, for the purposes of this study, data analysis was not performed for the inclusion criteria of the participants’ current clinical ladder. The findings only present the various attributes of HD nurses. It might be meaningful to recruit participants from different clinical ladders to realize how different clinical ladder affects cultural sensitivity of care HD patients in the future.

## Conclusions

The study highlighted the multifaceted aspects of the culturally sensitive care and experiences of HD nurses, as well as the nurses’ understanding of the cultural diversity of HD patients. The findings were based on the cultural perspectives of the interviewees while considering the diverse cultural backgrounds of the HD patients. These findings provide valuable insights for informing clinical practices aimed at addressing the cultural care needs of patients, ultimately contributing to an improved quality of clinical care.

## Data Availability

The data that support the findings of this study are available from the corresponding author upon reasonable request.
